# IoT-Cloud, VPN, and Digital Twin-Based Remote Monitoring and Control of a Multifunctional Robotic Cell in the Context of AI, Industry, and Education 4.0 and 5.0

**DOI:** 10.3390/s24237451

**Published:** 2024-11-22

**Authors:** Adrian Filipescu, Georgian Simion, Dan Ionescu, Adriana Filipescu

**Affiliations:** 1 Department of Automation, “Dunărea de Jos” University of Galați, 800008 Galați, Romania; georgian.simion@ugal.ro (G.S.); dan.ionescu@ugal.ro (D.I.); adriana.filipescu@ugal.ro (A.F.); 2 Doctoral School of Fundamental Sciences and Engineering, “Dunărea de Jos” University of Galați, 800008 Galați, Romania

**Keywords:** IoT, cloud, VPN, DT, AR, VR, control, MRC, AI, ML, industry, education 4.0/5.0

## Abstract

The monitoring and control of an assembly/disassembly/replacement (A/D/R) multifunctional robotic cell (MRC) with the ABB 120 Industrial Robotic Manipulator (IRM), based on IoT (Internet of Things)-cloud, VPN (Virtual Private Network), and digital twin (DT) technology, are presented in this paper. The approach integrates modern principles of smart manufacturing as outlined in Industry/Education 4.0 (automation, data exchange, smart systems, machine learning, and predictive maintenance) and Industry/Education 5.0 (human–robot collaboration, customization, robustness, and sustainability). Artificial intelligence (AI), based on machine learning (ML), enhances system flexibility, productivity, and user-centered collaboration. Several IoT edge devices are engaged, connected to local networks, LAN-Profinet, and LAN-Ethernet and to the Internet via WAN-Ethernet and OPC-UA, for remote and local processing and data acquisition. The system is connected to the Internet via Wireless Area Network (WAN) and allows remote control via the cloud and VPN. IoT dashboards, as human–machine interfaces (HMIs), SCADA (Supervisory Control and Data Acquisition), and OPC-UA (Open Platform Communication-Unified Architecture), facilitate remote monitoring and control of the MRC, as well as the planning and management of A/D/R tasks. The assignment, planning, and execution of A/D/R tasks were carried out using an augmented reality (AR) tool. Synchronized timed Petri nets (STPN) were used as a digital twin akin to a virtual reality (VR) representation of A/D/R MRC operations. This integration of advanced technology into a laboratory mechatronic system, where the devices are organized in a decentralized, multilevel architecture, creates a smart, flexible, and scalable environment that caters to both industrial applications and educational frameworks.

## 1. Introduction

The paper proposes the design and implementation of remote or local monitoring and control of a laboratory mechatronics system based on an IoT-cloud, VPN, and DT approach. The mechatronic system is the MRC, equipped with an industrial robotic manipulator, ABB 120 IRM, which performs the flexible assembly of two workpieces, each consisting of five components. The system can also perform disassembly and replacement allowing the parts to be recovered, possibly for reuse or resale. If the assembled workpiece is completely compromised, it is returned to the MRC on the conveyor belt for complete disassembly or replacement of parts [1,2,3,4]. The ABB 120 IRM integrated into the MRC performs intricate A/D/R tasks using data from IoT sensors that monitor component positioning, alignment, and force applied during joining. The system adapts in real time to any variances, using data-driven insights from cloud-based AI (ML) models. Through the equipment used as edge IoT devices and the software packages involved, the whole system aims to respond to Industry/Education 4.0 and 5.0. Edge IoT devices, such as a wireless router, PLC, embedded computer, image processor, ABB IRM Controller, communication devices, and servo drivers, are connected in a local network, which are, in turn, connected to the Internet via WAN [4,5]. Additionally, for IoT integration it is necessary to equip the MRC with IoT sensors and cameras to capture real-time operational data during assembly, disassembly, and part replacement tasks. IoT gateways are used to aggregate sensor data and transmit it securely to the cloud for real-time analysis and control actions. In the future, we intend to develop a cloud platform to host data, analytics, and control applications and use cloud-based applications for visualizing the status of assembly, disassembly, or replacement operations, including task progression, error reporting, and predictive maintenance alerts. All of these enable remote monitoring, real-time feedback, and decision-making capabilities. The role of the VPN connection is to provide secure, encrypted communication between the MRC and an authorized remote user. The VPN ensures that data between the MFC, cloud platform, and remote user are protected from cyber threats [6,7,8].

The DT is a virtual replica of the MRC system, including the ABB 120 IRM and its interactions with the A/D/R tasks. It mirrors the real-world processes, allowing real-time monitoring, simulation, and task optimization. The assignment, planning, and execution of tasks in simulated mode, consisting of logical schemes connected to hardware components, and visual signaling of the current operation performed by the ABB IRM, represent a concept of AR. The modeling of A/D/R operations through STPN, the formalization and simulation of the models, represents the VR counterpart for the real-time operations of the MRC. AR adds an interactive layer of digital information into the physical world, which can greatly enhance task planning, troubleshooting, and operator assistance during A/D/R tasks. AR can provide step-by-step instructions for A/D/R tasks, enhancing accuracy and speed by guiding the user through complex procedures in real time. Remote users can customize AR to visualize the status of the MRC and provide support to on-site personnel by seeing what they see and guiding them through tasks remotely. VR allows operators to enter a fully immersive virtual environment that simulates the MRC’s tasks and workflows. Coupling VR with STPN models enhances the user experience by providing a formal model for analyzing and optimizing tasks. STPN simulation within VR environments can be used to map out and test task flows for the ABB 120 IRM [4,8,9,10,11,12,13]. This method ensures that A/D/R tasks follow the most efficient sequence while considering time constraints. Users can experience the entire A/D/R process virtually, identifying potential inefficiencies before deploying on the physical MRC. The monitoring signals acquired from the PLC must correspond to those obtained from the virtual world.

A SCADA system is integrated into the system for real-time control and automation. SCADA gathers data from IoT sensors and actuators on the ABB 120 IRM and other devices to provide real-time feedback to the operators. The SCADA system allows users to monitor and control the ABB IRM’s movements, the sequencing of A/D/R tasks, and the status of connected devices (e.g., conveyors, pick-and-place systems). SCADA interfaces provide visualizations of the MRC’s status, showing real-time data such as component positions, actuator states, and task progression. Users can use this data to make informed decisions. The SCADA system can trigger automatic actions based on predefined conditions or operator inputs, ensuring that the MRC operates autonomously within set safety and performance parameters [10,11,12,13,14,15,16,17,18,19,20,21,22].

Several HMIs are designed, allowing users to interact, remotely or locally, with the MRC, access key metrics, and manually control it when needed. The HMIs provide real-time feedback from SCADA, DT, IoT sensors, and detailed information about the MRC status, including the workpiece’s A/D/R progress. In the future, the HMIs will be enhanced with AI (ML)-powered decision support, offering recommendations for task sequencing, maintenance schedules, or component adjustments based on the system status [4].

OPC-UA is integrated to ensure standardized and secure communication between various devices in the MRC. It enables interoperability between different systems such as the ABB 120 IRM, A/D/R stations, IoT sensors, PLC, embedded computer, cameras, video processing unit (VPU), IoT gateway, conveyor driver, and cloud-platform. OPC-UA acts as a bridge between the local control systems (SCADA, HMIs) and the cloud-based infrastructure, facilitating smooth data flow, A/D/R process coordination, and real-time communication. HMI-type SCADA systems are designed both for initialization and configuration and to work remotely and locally for monitoring and control [5,6,7]. Through a specialized communication device, instantaneous and time-horizon electrical data will be acquired from sensors, stored in the embedded computer or in the cloud, useful for developing an adaptive ML for the prevention of breakdowns and maintenance. Thus, the system has robust operation, at the same time ensuring data protection and inviolability [4].

The rest of the paper is organized into six sections. The hardware structure of the A/D/R MRC for remote or local monitoring and control, with a multilevel architecture based on IoT edge devices, LAN, WAN networking, cloud, and VPN connections, is presented in Section 2. In Section 3, digital twin’s virtual-world counterpart of A/D/R MRC, together with task planning as AR and STPN models, formalism and simulation as VR— all of these for assembly, disassembly, and cylinder replacement—are presented in Section 3. IoT-cloud and VPN remote monitoring and control, together with remote or local initialization and selection via A/D/R HMIs, cloud- and embedded-computer-based data storage and analytics, machine learning for adaptive control, optimization, predictive maintenance, type-3 fuzzy logic, and fractional-order learning in the context of AI, are presented in Section 4. Several remarks about the approach and results and how they fit into the context of AI (ML), Industry and Education 4.0 and 5.0, can be found in Section 5, Discussion. Final remarks, paper contributions, and future research are stated in Section 6, Conclusions.

## 2. Hardware Structure of A/D/R MRC

### 2.1. IoT Edge Devices and LAN-Profinet/LAN-Ethernet/WAN-Ethernet and Networking

**Figure 1:**
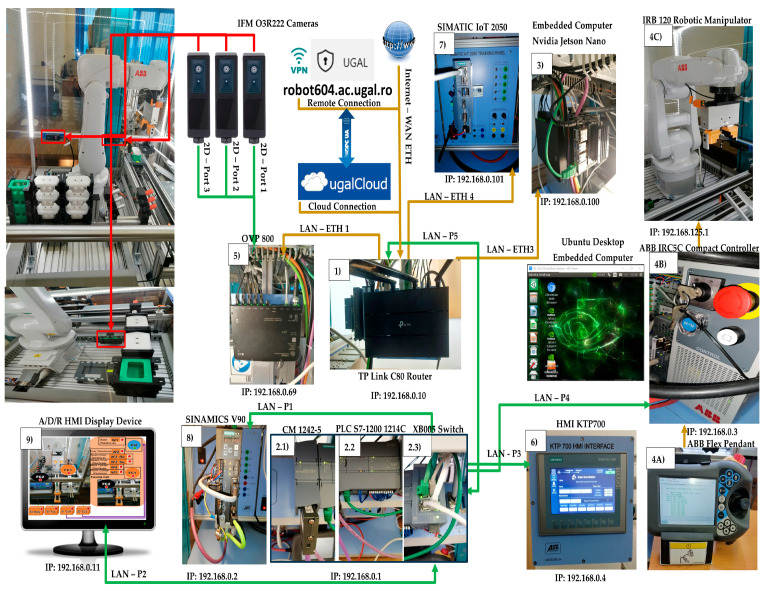
IoT edge devices and LAN/WAN networking.

In Figure 1, all the devices connected to the LAN are displayed, each with its own unique IP address. (1) The Wi-Fi router (TP-Link Archer) has the role of Wi-Fi network management, ensures connection to local networks, LAN-Profinet (green), and LAN-Ethernet (cyan), and to the Internet via WAN-Ethernet (cyan) and OPC-UA. (2) SIEMENS PLC 1214C DC/DC/DC, as the HMI’s main control unit, allows all other devices to communicate with it, manages all signals (on sensors or from communication), and internal variables, which are used by remote and local SCADA systems. Remote and local SCADA systems use PLC’s internal variables, send/receive data to/from IFM devices (3D cameras that provide images and information about the number of parts in the storage warehouses before and after a handling operation), and are programmed in TIA V17. (3) The embedded computer NVIDIA Jetson Nano has a Virtual Network Computing (VNC) server running on it, and VNC Viewer is enabled for remote connection. The Jetson Nano has access to all the other devices, a web browser for the Node-RED interface, has an installed docker environment, and docker images. The Jetson Nano embedded computer can run the same docker image as the OVP800 for camera management, allows Node-RED programming and testing, and docker development. (4A) The ABB touch pendant, (4B) ABB IRC5C controller, and (4C) the 6-DOF ABB 120 IRM, together with the IO–Interface: 192.168.0.3 (PLC signals), and service 192.168.125.1 (Robot Studio Communication) execute the actions configured by the user on remote and local HMI SCADA dashboards, send/receive data to/from PLC, and are programmed in Robot Studio. (5) The video processing unit (VPU), OVP 800, ensures artificial vision management, sends/receives data to/from PLC, and controls the 3 cameras via ROS2 Humbles using the IFM3D_ROS2 library. The OVP 800 runs ROS2 Humbles for cameras management, parts detection algorithm, PLC communication, and WebSocket server (for Node-RED communication). (6) The local SCADA system HMI KTP 700 allows the operator to locally control FRC and send/receive data to/from the PLC. (7) The SIEMENS IOT2050 is used as the remote SCADA system, sends/receives data to/from the PLC, video streams from IFM cameras, stores energy records, and can replicate the HMI KTP 700 interfaces. (8) The SIEMENS S INAMICS V90 is the conveyor control unit, sends/receives data to/from the PLC, and is programmed in TIA V17. (9) The local HMI display device. (2.3) The communication adapter XB005 Switch, attached to the S7-1214 PLC, is used for connecting the following devices, via LAN, to digital ports: to P1, SINAMICS V90; to P3, secondary SCADA system, HMI-KTP 700; to P4, ABB controller; to P5, the Wi-Fi router, and to P2, the A/D/R local HMI display device (2.1) The communications module SIMATIC CM 1242-5, attached to the S7-1214 PLC, is used for the connection of the SIMATIC S7-1214 PLC to PROFIBUS (DP slave module). The MRC has 6 warehouses for storing three components each: Base (Pallet) warehouse (W1); Body warehouse (W2); Round Cover (Top_Rd) warehouse (W3_Rd), Square Cover (Top_Sq) warehouse (W3_Sq); Metal cylinder warehouse (W4); and Plastic cylinder warehouse (W5). Three IFM O3R222 cameras, for detecting the number of parts in the warehouse, are as follows: the Base detection camera is connected to port 1 of the VPU-OVP800; the Body and Top detection camera is connected to port 2 of the VPU-OVP800; and the camera for the detection of metal and plastic cylinders is connected to port 3 of the VPU-OVP800.

The MRC is equipped with a conveyor belt for transporting the workpiece to the left and right entrances/exits. At the two inputs and outputs, there are inductive sensors that signal the presence of the workpiece according to the metal markers on the Base (Pallet). Thus, the MRC can be interconnected with other mechatronics stations that operate A/D/R on the same types of workpieces and parts (e.g., 6-workstation Mechatronics line, Hera and Horstman [4]).

### 2.2. Cloud and VPN-Based Monitoring and Control Multilevel Architecture

**Figure 2:**
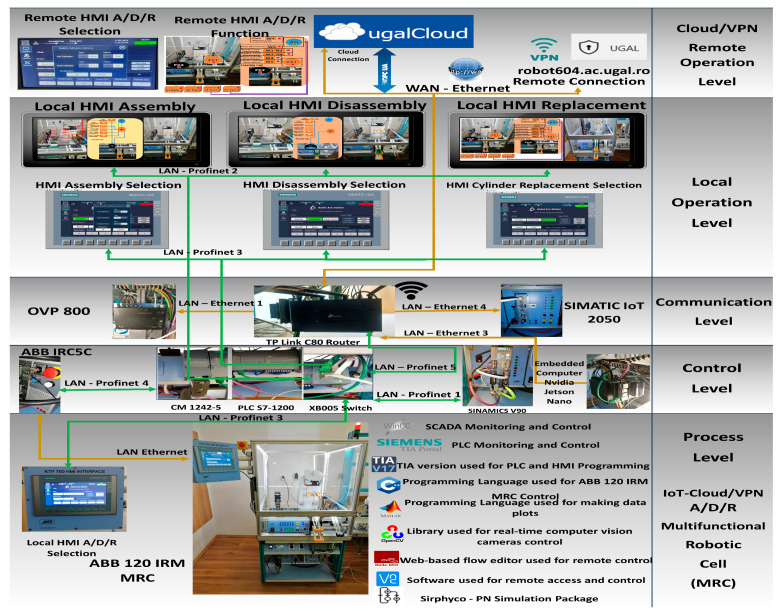
Cloud- and VPN-based remote monitoring and control multilevel architecture.

The flexible A/D/R operations performed by MRC consist of the assembly of two workpieces (WP1, WP2), disassembly, and replacement of cylinders with the recovery of parts for reuse [4]. The control structure, shown in Figure 2, is multilevel as follows: (1) Cloud/VPN remote operation level, with SCADA, remote HMI A/D/R, and remote HMI selection A/D/R for remote monitoring and control of assembly, disassembly, and replacement. (2) Local operation level, with SCADA, local HMI A/D/R, and local HMI selection A/D/R for local monitoring and control of assembly, disassembly, and replacement. (3) Communication level with video processing unit (VPU) OVP 800, Wi-Fi router, TP-Link Archer, and SIMATIC IOT2050 as the primary SCADA edge device that connects the various subsystems to the display devices or to the cloud. (4) Control level with the ABB IRC5C controller of ABB 120 IRM, PLC S7-1200 as main control unit, together with the Switch Ethernet Simatic SCALANCE XB005, SIEMENS CM 1242-5, and SINAMICS V90 for conveyer control, and NVIDIA Jetson Nano, a small, powerful computer for embedded applications that delivers the power for remote SCADA monitoring and control. (5) Process level with the ABB 120 IRM with an electric gripper that ensures the handling of parts inside the cell, and HMI KTP 700 as the local HMI selection of A/D/R MRC. Next to the MRC, the software packages used are listed along with their usage.

## 3. Digital Twin’s Virtual World Counterpart of A/D/R MRC

The virtual world, as the counterpart of the digital twin of the A/D/R MRC, has two components. The first one is the augmented reality associated with the planning of tasks related to each of the functionalities (assembly, disassembly, and replacement of cylinders). The second is the virtual reality associated with the handling of displacements performed by ABB IRM [23,24,25,26,27]. The task planning is conceived as an AR implemented as a flow of Node-RED functions ([28]), which is transposed into an HMI in which the operations performed by ABB IRM and the tasks in the organization chart are executed synchronously (using PLC signals). The execution of a task corresponds to a monitoring signal, which in the organizational chart is signaled by lighting the spotlight.

**Figure 3:**
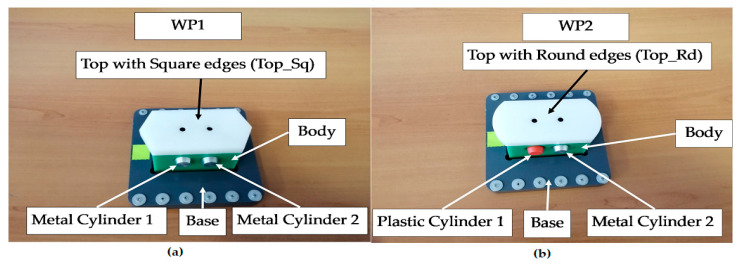
(**a**,**b**) The parts of the workpieces, WP1 and WP2. (**a**) WP1 with Top_Sq; (**b**) WP2 with Top_Rd.

To model and simulate the virtual reality of each MRC functionality, the Synchronized Timed Petri Nets tool is used. Starting from the linear and angular displacement limitations of ABB IRM, based on the times required for handling the parts and transporting the workpiece on the conveyor belt, the STPN models related to the assembly, disassembly, and replacement of cylinders were released. In the STPN models, the states in red are control states associated with the control functions of the decision actions, which are states that trigger a transition when they receive a token. The states in brown or gray correspond to the pick-and-place actions and receive a token after the end of the transition. Yellow states correspond to monitoring actions and receive a token after the handling or transport transition has been completed. There is also a state in green, only for assembling and replacing cylinders, which receives a token when an assembled part is delivered or with replaced cylinders. There are also three states in red, synchronization signals that receive a token when one of the functionalities has been completed, conditioning the start of another. The simulation of an STPN model is performed in the Sirphyco package [8,9,10,11,12,13] and [29]. The MRC performs each functionality—assembly, disassembly or replacement of cylinders—for two workpieces, called WP1 or WP2; the difference between them is shown in Figure 3.

### 3.1. Task Planning for Assembly as Augmented Reality

**Figure 4:**
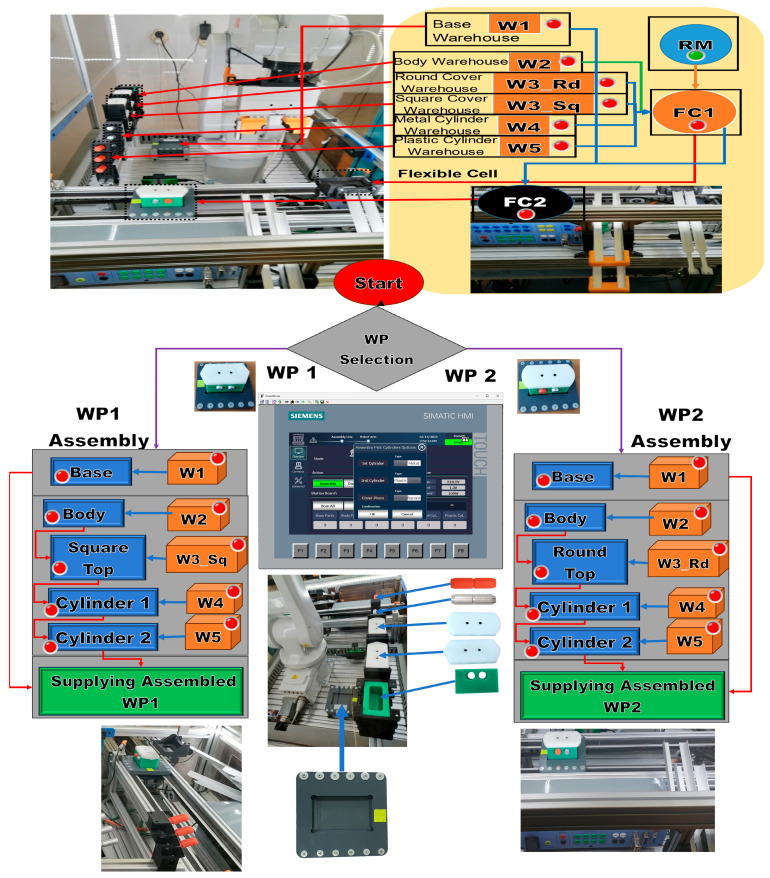
Node-RED assembly task planning as augmented reality.

After the assembly is completed, the assembled workpiece is delivered on the conveyor belt at the right exit of the MRC, a location that is also used for the workpiece arriving for disassembly or cylinder replacement. The parts to be assembled are as follows: (1) Base (Pallet), (2) Body, (3) Top with square edges (WP1) or Top with rounded edges (WP2), (4) the left hole of the Body as the first cylinder (Cylinder1), and (5) the right hole of the Body as the second cylinder (Cylinder2), both, metal and plastic cylinders. First, the Base is positioned on the conveyor belt, called FC2; then the rest of the product is assembled in a separate location in the MRC, called FC1. Afterward, it is moved by the ABB IRM onto the Base, on FC2. Finally, WP1 or WP2 is transferred, along the conveyor belt, to the left-side exit of the MRC. In Figure 4, the locations, FC1 and FC2, where the partial (Body, Top_sq or Top_rd, Cyl1 and Cyl2) and total assemblies are made by transferring and placing the workpiece with the ABB IRM from FC1 to FC2 on the Base, are marked. Assembly task planning is conceived as AR implemented as a flow of Node-RED functions that is transposed to the remote or local HMI, where the assembly operations, performed by the ABB IRM, run synchronously between the MRC and the task flowchart, as shown in Figure 4.

### 3.2. Task Planning for Disassembly as Augmented Reality

**Figure 5:**
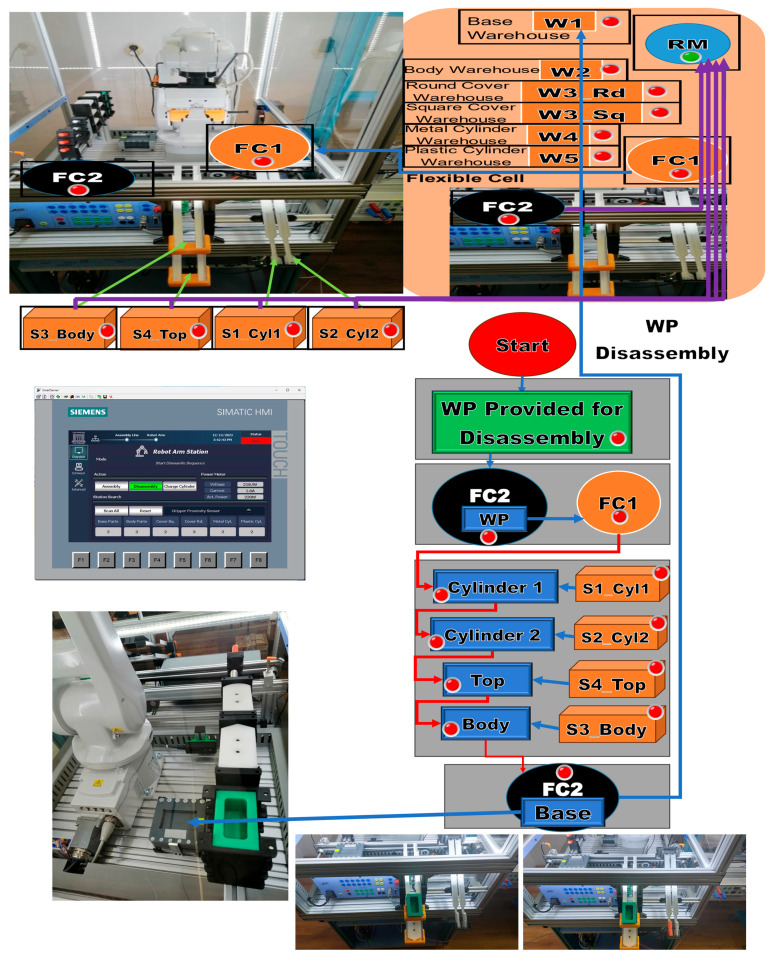
Node-RED disassembly task planning as augmented reality.

WP1 or WP2 is available for disassembly on the conveyor belt at the right entrance of the MRC. It is transported by conveyor to the FC2 location. The ABB IRM takes the partially assembled workpiece and places it in the location of FC1. Here, the disassembly is done by the ABB IRM by means of the gripper accessories, in order to push Cylinder1 and Cylinder2 by sliding on the inclined chutes S1_Cyl1 and S2_Cyl2. Then, grab and manipulate the Top by sliding it onto S4_Top. In the same way, it grabs and manipulates the Body, leaving it by sliding on S3_Top. The last operation performed by the ABB IRM is taking the Base from FC2 and placing it in warehouse W1. Similar to assembly, task planning for disassembly is conceived as augmented reality implemented as a flow of Node-RED functions that is transposed to the remote or local HMI where the disassembly operations, performed by ABB IRM, run synchronously between the MRC and the task flowchart, as shown in Figure 5.

### 3.3. Task Planning for Replacing Cylinders as Augmented Reality

**Figure 6:**
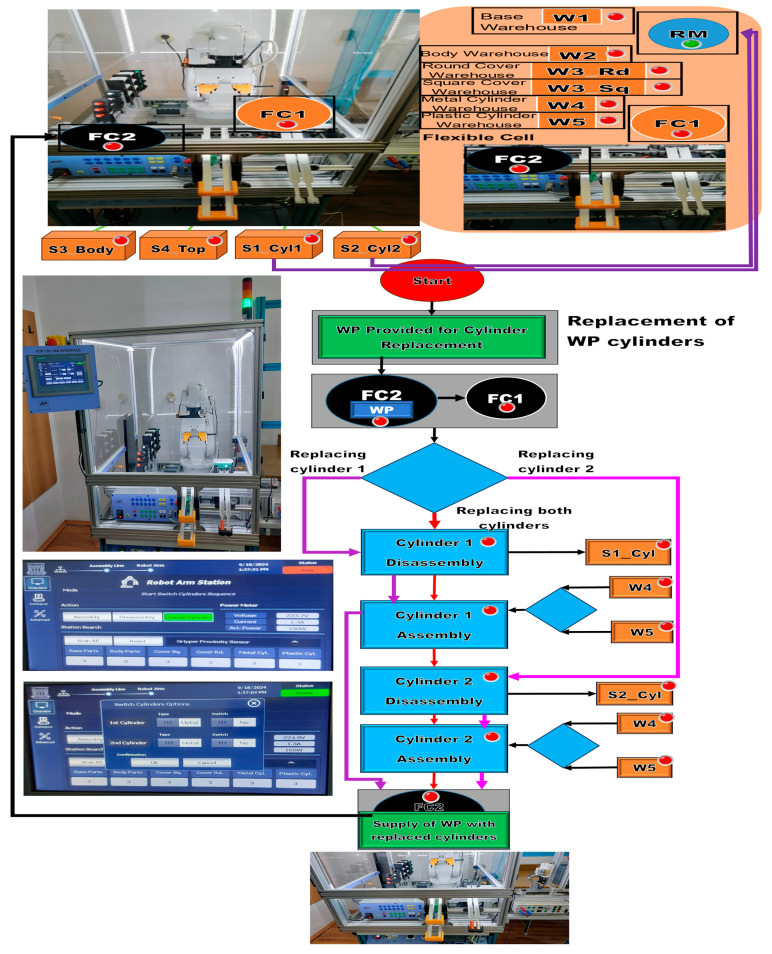
Node-RED cylinder replacement task planning as augmented reality.

WP1 or WP2 is available for the replacement of cylinders on the conveyor belt at the right entrance of the MRC. It is transported by conveyor to the FC2 location. The ABB IRM takes the partially assembled workpiece and places it at the location of FC1. The sequence of operations when changing the cylinders is as follows: If in the remote or local HMI selection the change of a single cylinder (Cylinder 1 or 2) has been selected, then the corresponding cylinder is disassembled and pushed onto the inclined chute S1_Cyl1 or S2_Cyl2. Then, the ABB IRM takes the metal or plastic cylinder from W4 or W5 (corresponding to the HMI option) and assembles it in the corresponding hole. If it was decided that both cylinders need to be changed, the operations of changing a single cylinder are repeated in the order of cylinder 1 followed by cylinder 2. Afterward, it is moved by the ABB IRM onto the Base, on FC2. Finally, WP1 or WP2, with the cylinder replaced, is transferred along the conveyor belt to the right-side exit of the MRC. Like assembly and disassembly, task planning for cylinder replacement is conceived as AR implemented as a flow of Node-RED functions that is transposed to the remote or local HMI where the replacement operations, performed by ABB IRM, run synchronously between the MRC and the organization chart tasks, as shown in Figure 6.

### 3.4. STPN Model, Formalism and Simulation for Assembly as Virtual Reality

**Figure 7:**
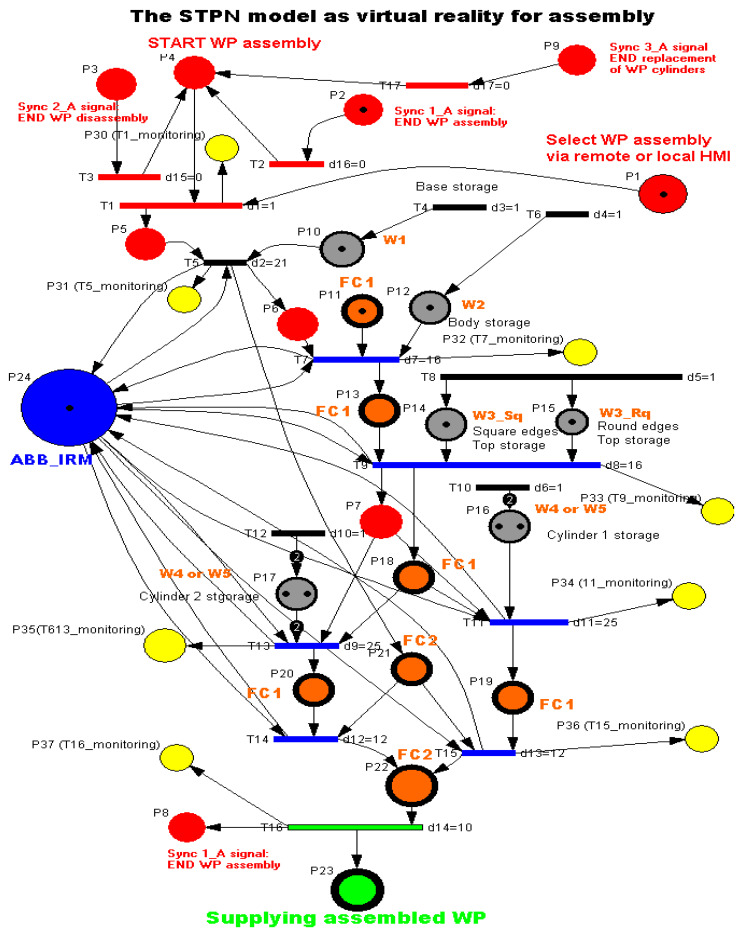
The STPN model as VR for assembly.

Following the task planning, the STPN model was developed for the assembly of WP1 or WP2, which involves taking parts from the warehouses, handling and assembling them in FC1 and FC2, respectively, and transporting the assembled workpiece on the conveyor to the right exit of the FRC, all this with the corresponding time durations. Three synchronization signals from the sensors are required to signal that the previous assembly, disassembly, or cylinder replacement has ended [4,5,6,7]. The STPN model is shown in Figure 7.

The STPN model is defined by

(1) $$STPN_{A}=\langle \begin{array}{ccc} TPN_{A}, & E, & Sync \end{array}\rangle ,$$

where

(2) $$TPN_{A}=\langle P_{A},\;T_{A},\;Pre_{A},Post_{A},\;m_{0_{A}},\;tempo_{A}\rangle .$$

The elements of the $TPN_{A}$ from (2) are

- P_A_ is the place set partitioned into
  (3) $$P_{A}=\left\{P_{control_{A}},P_{assembly_{A}},P_{monitoring_{A}}\right\},$$
  where
  (4) $$P_{{\mathrm{control}}_{A}}={\left\{P_{i_{A}}\right\}}_{i_{A}\in \left\{1,2,3,4,5,6,7,8,9\right\}}$$
  represents the state set associated with control functions of the decision actions,
  (5) $$P_{{\mathrm{assembly}}_{A}}={\left\{P_{j_{A}}\right\}}_{j_{A}=\bar{10,24}}$$
  represents the set of the discrete places modeling the flexible assembly operations for the two work pieces, WP1 and WP2, and
  (6) $$P_{{\mathrm{monitoring}}_{A}}={\left\{P_{j_{A}}\right\}}_{j_{A}=\bar{30,37}}$$
  represents the set of the states for monitoring the successive assembly actions for WP1 or WP2.
- T_A_ is the transitions set partitioned into
  (7) $$T_{A}=\left\{T_{assembly_{A}},T_{assembled_{A}}\right\}$$
  where
  (8) $$T_{assembly_{A}}={\left\{T_{i_{A}}\right\}}_{i_{A}=\left\{1,2,3,4,5,6,7,8,9,10,11,12,13,14,15,17\right\}}$$
  is the set of discrete transitions for the two-workpiece (WP1, WP2) assembly, and
  (9) $$T_{assembled_{A}}=\left\{T_{16_{A}}\right\}$$
  is the transition associated with the conveyor transport of the assembled workpiece at the right exit of the MRC.

For WP1 or WP2 assembly on the MRC with the ABB 120 IRM, the monitoring places in set (6) monitor the transitions in set (8) as follows: P30 (monitors T1), P31 (monitors T5), P32 (monitors T7), P33 (monitors T9), P34 (monitors T11), P35 (monitors T13), P36 (monitors T15), and P37 (monitors T17).

- $Pre_{A}:P_{A}\times T_{A}\to Q_{{+}_{Apre}}$ is the input incidence function.
- $Post_{A}:P_{A}\times T_{A}\to Q_{{+}_{Apost}}$ is the output incidence function.
- $m_{0_{A}}$ is the initial marking of the STPN corresponding to the initial state of the modeled process.
- $tempo_{A}:T_{A}\to Q_{{+}_{AT}}\cup \left\{0\right\}$ is a function that defines the timings associated with the transitions.
  (10) $$E=\left\{Ed^{1},Ed^{2},Ed^{3}\right\}\cup \left\{e\right\}$$
  is the set of external events.

The Sync application in Definition (1) is a function from the set of discrete assembly transitions to the set of external events joined with the neutral element e,

(11) $$Sync\;:\left\{T_{2},\;T_{3},\;T_{17}\right\}\to \left\{Ed^{1},\;Ed^{2},Ed^{3}\right\}\cup \left\{e\right\},$$

(12) $$Sync\;1\_A:T_{2}\to \left\{Ed^{1}\right\},$$

(13) $$Sync\;2\_A:T_{3}\to \left\{Ed^{2}\right\},$$

(14) $$Sync\;3\_A:T_{17}\to \left\{Ed^{3}\right\}$$

$Ed^{1}=Sync1\_Asignal$ is the synchronization signal for: END WP assembly of WP1 or WP2.

$Ed^{2}=Sync2\_Asignal$ is the synchronization signal for: END WP disassembly of WP1 or WP2.

$Ed^{3}=Sync3\_Asignal$ is the synchronization signal for: END replacement of WP cylinders of WP1 or WP2.

**Figure 8:**
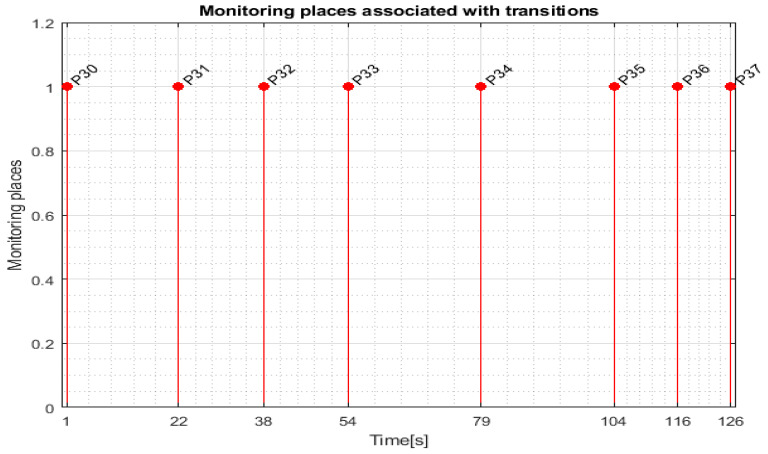
Sirphyco simulation of the STPN model for the assembly.

Transition monitoring states obtained by the STPN model simulation in Sirphyco, for the assembly processes of WP1 or WP2, are presented in Figure 8 [29,30]. As a result of the simulation of the STPN model, it can be seen in Figure 8 that the monitoring states receive tokens at a time interval corresponding to the initiation of the assembly function, together with the time interval associated with the current transition.

### 3.5. STPN Model, Formalism, and Simulation for Disassembly as Virtual Reality

**Figure 9:**
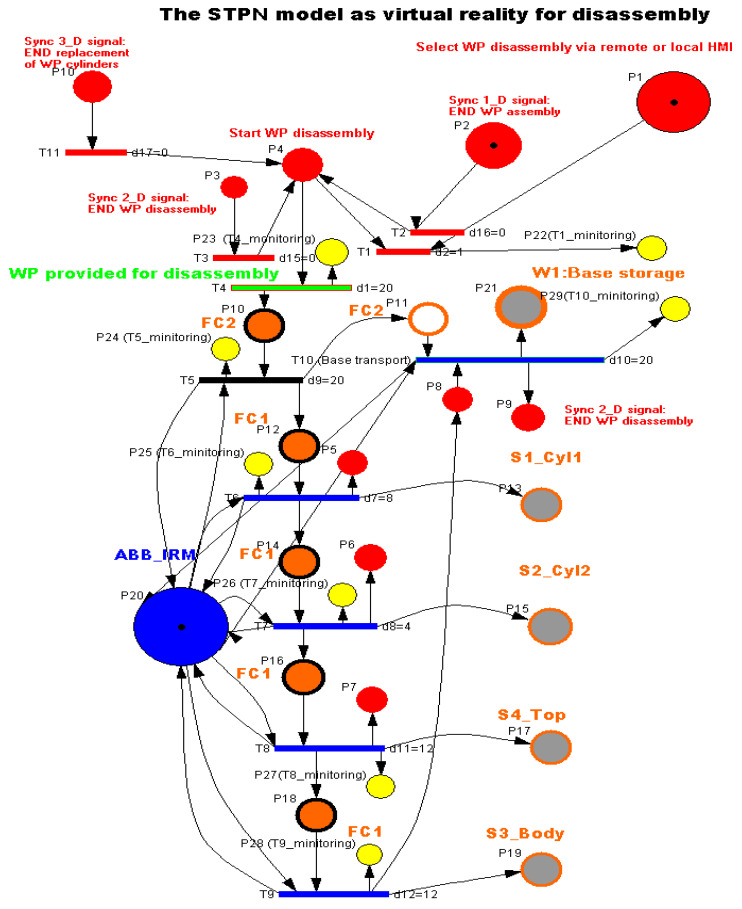
STPN model as VR for disassembly.

WP1 or WP2 is available for disassembly on the conveyor belt at the right entrance of the MRC. It is transported by the conveyor to the FC2 location. The ABB IRM takes the partially assembled workpiece (the part above the Pallet) and places it in the location of FC1. Here, the disassembly is performed by the ABB IRM by means of gripper accessories. The parts are disassembled by pushing them onto the slide chutes. At the end, the pallet is moved from FC2 to W1. The STPN model, with the corresponding transition time durations, is presented in Figure 9 [3,4,5,6]. The same three synchronization signals coming from the sensors are needed to signal that the previous assembly, disassembly, or cylinder replacement has been completed.

The STPN model for the disassembly process is a triplet.

(15) $$STPN_{D}=\left\langle \begin{array}{ccc} TPN_{D}, & E, & Sync \end{array}\right\rangle ,$$

where TPN is the timed Petri net model, E is a set of external events, and Sync is an application from the set of transitions to that of external events.

The TPN is a sextuplet:

(16) $$TPN_{D}=\left\langle P_{D},\;T_{D},\;Pre_{D},\;Post_{D},\;m_{0_{D}},\;tempo_{D}\right\rangle .$$

The elements of the $TPN_{D}$ from (16) are

- P_D_ is the set of places set, partitioned into
  (17) $$P_{D}=\left\{P_{control_{D}},P_{disassembly_{D}},P_{monitoring_{D}}\right\},$$
  where
  (18) $$P_{\mathrm{contro}l_{D}}={\left\{P_{i_{D}}\right\}}_{i_{D}=\bar{1,9}}$$
  represents the set of states, associated with the control functions of the decision actions,
  (19) $$P_{\mathrm{disassembl}y_{D}}={\left\{P_{j_{D}}\right\}}_{j_{D}=\bar{10,21}},$$
  represents the set of discrete places modeling the flexible disassembly operations for the two workpieces (WP1 and WP2), and
  (20) $$P_{\mathrm{monitorin}g_{D}}={\left\{P_{j_{D}}\right\}}_{j_{D}=\bar{22,29}},$$
  represents the state set associated with the monitoring of the successive disassembly actions for WP1 or WP2.
- T_D_ is the transitions set partitioned into
  (21) $$T_{D}=\left\{T_{ready\;for\;disassembly_{D}},T_{disassembly_{D}}\right\}$$
  where
  (22) $$T_{ready\;for\;disassembly_{D}}=\left\{T_{4_{D}}\right\},$$
  is the set of discrete transitions associated with WP delivered for disassembly,
  (23) $$T_{disassembly_{D}}={\left\{T_{i_{D}}\right\}}_{i_{D}\in \left\{1,5,6,7,8,9,10\right\}},$$
  is the set of the discrete transitions for the two-workpiece (WP11 or WP2) disassembly.

For WP1 or WP2 disassembly on the MRC with ABB 120 IRM, the monitoring places in set (20) monitor the transitions in set (23) as follows: P22 (monitors T1), P23 (monitors T4), P24 (monitors T5), P25 (monitors T6), P26 (monitors T7), P27 (monitors T8), P28 (monitors T9), and P29 (monitors T10).

- $Pre_{D}:P_{D}\times T_{D}\to Q_{{+}_{Dpre}}$ is the input incidence function.
- $Post_{D}:P_{D}\times T_{D}\to Q_{{+}_{Dpost}}$ is the output incidence function.
- $m_{0_{D}}$ is the initial marking of the STPN corresponding to the initial state of the modeled process.
- $tempo_{D}:T_{D}\to Q_{{+}_{DT}}\cup \left\{0\right\}$ is a function that defines the timings associated with the transitions.
  (24) $$E=\left\{Ed^{1},Ed^{2},Ed^{3}\right\}\cup \left\{e\right\},$$
  is the set of external events.

The Sync application in Definition (1) is a function from the set of discrete disassembly transitions to the set of external events joined with the neutral element e,

(25) $$Sync\;:\left\{T_{2},\;T_{3},\;T_{11}\right\}\to \left\{Ed^{1},\;Ed^{2},Ed^{3}\right\}\cup \left\{e\right\},$$

(26) $$Sync\;1\_D:T_{2}\to \left\{Ed^{1}\right\},$$

(27) $$Sync\;2\_D:T_{3}\to \left\{Ed^{2}\right\},$$

(28) $$Sync\;3\_D:T_{11}\to \left\{Ed^{3}\right\}$$

- $Ed^{1}=Sync\;1\_Dsignal$ is the synchronization signal for: END WP assembly of WP1 or WP2.
- $Ed^{2}=Sync2\_Dsignal$ is the synchronization signal for: END WP disassembly of WP1 or WP2.
- $Ed^{3}=Sync3\_Dsignal$ is the synchronization signal for: END replacement of WP cylinders of WP1 or WP2.

**Figure 10:**
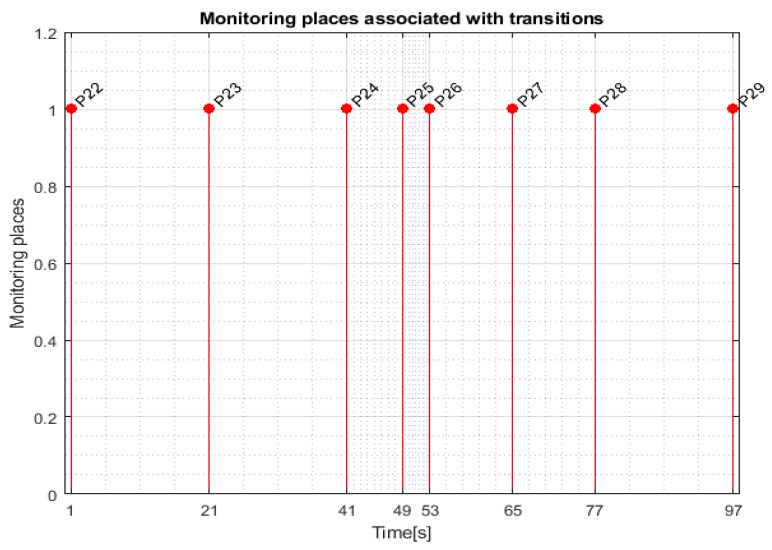
Sirphyco simulation of STPN model for the disassembly.

Transition monitoring states obtained by the STPN model simulation in Sirphyco, for the disassembly process of the WP1 or WP2, are presented in Figure 10. As a result of the simulation of the STPN model, it can be seen in Figure 10 that the monitoring states receive tokens at a time interval corresponding to the initiation of the disassembly function, together with the time interval associated with the current transition.

### 3.6. STPN Model, Formalism, and Simulation for Cylinder Replacement as Virtual Reality

WP1 or WP2 is available for replacement of cylinders on the conveyor belt at the right entrance of the MRC. It is transported by the conveyor to the FC2 location. The ABB IRM takes the partially assembled workpiece and places it in the location of FC1. If the change of a single cylinder (cylinder 1 or 2) has been selected, then the corresponding cylinder is disassembled and pushed onto the inclined chute S1_Cyl1 or S2_Cyl2. Then, ABB IRM takes the metal or plastic cylinder from W4 or W5 and assembles it in the corresponding hole. If it was decided to change both cylinders, then the operations of changing a single cylinder are repeated in the order of cylinder 1 followed by cylinder 2.

**Figure 11:**
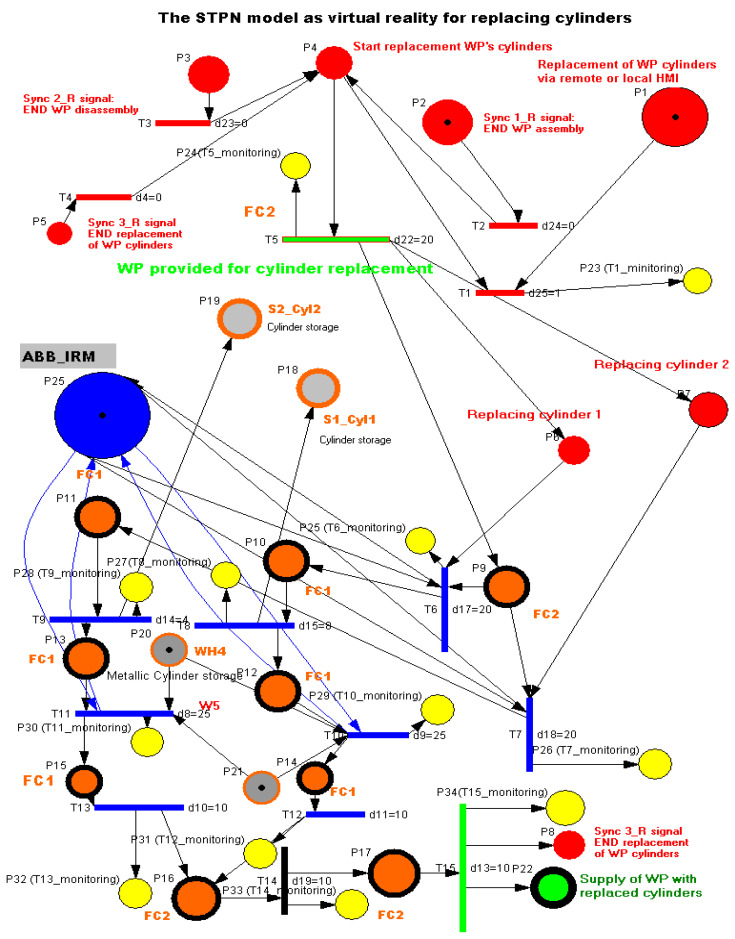
STPN model as VR for replacing cylinders.

After it is moved by the ABB IRM onto the Base, on FC2. Finally, WP1 or WP2, with the cylinder replaced, is transferred along the conveyor belt to the right-side exit of the MRC. The STPN model for cylinder replacement, together transition time durations, are presented in Figure 11. The same three synchronization signals coming from the sensors are needed to signal that the previous assembly, disassembly, or cylinder replacement has been completed.

The STPN is defined by

(29) $$STPN_{R}=\left\langle \begin{array}{ccc} TPN_{R}, & E, & Sync \end{array}\right\rangle ,$$

(30) $$TPN_{R}=\left\langle P_{R},\;T_{R},\;Pre_{R},\;Post_{R},\;m_{0_{R}},\;tempo_{R}\right\rangle .$$

The elements of the $TPN_{R}$ from (15) are

- P_R_ is the set of places, partitioned into
  (31) $$P_{R}=\left\{P_{control_{R}},P_{replacing_{R}},P_{monitoring_{R}}\right\},$$
  where
  (32) $$P_{\mathrm{contro}l_{R}}={\left\{P_{i_{R}}\right\}}_{i_{R}=\bar{1,8}},$$
  represents the set of states, associated with the control functions of the decision actions,
  (33) $$P_{\mathrm{replacin}g_{R}}={\left\{P_{j_{R}}\right\}}_{j_{R}=\bar{10,23}},$$
  represents the set of discrete places modeling the flexible disassembly operations for the two workpieces (WP1 and WP2), and
  (34) $$P_{\mathrm{monitorin}g_{R}}={\left\{P_{j_{R}}\right\}}_{j_{R}=\bar{24,35}},$$
  represents the states set associated to the monitoring of the disassembly actions.
- T_R_ is the transition set partitioned into
  (35) $$T_{R}=\left\{T_{ready\;for\;replacing_{R}},T_{replacing_{R}},T_{replaced_{R}}\right\},$$
  where
  (36) $$T_{ready\;for\;replacing_{R}}=\left\{T_{5_{R}}\right\},$$
  is the set of discrete transitions associated with the WP delivered for disassembly,
  (37) $$T_{replacing_{R}}={\left\{T_{i_{R}}\right\}}_{i_{R}=\bar{5,14}},$$
  is the set of discrete transitions for cylinder replacement of the two workpieces.
  (38) $$T_{replaced_{R}}=\left\{T_{15_{R}}\right\},$$
  is the transition associated with the conveyor transport to the right exit of the MRC of the workpiece with cylinder replaced.

For replacing the cylinders of WP1 or WP2 on the MRC with the ABB 120 IRM, the monitoring places in set (34) monitor the transitions in set (37) as follows: P24 (monitors T1), P25 (monitors T5), P26 (monitors T6), P27 (monitors T7), P28 (monitors T8), P29 (monitors T9), P30 (monitors T10), P31 (monitors T11), P32 (monitors T12), P33 (monitors T13), P34 (monitors T14), P35 (monitors T15).

- $Pree_{R}:P_{R}\times T_{R}\to Q_{{+}_{Rpre}}$ is the input incidence function.
- $Post_{R}:P_{R}\times T_{R}\to Q_{{+}_{R\;post}}$ is the output incidence function.
- $m_{0_{R}}$ is the initial marking of the STPN corresponding to the initial state of the modeled process.
- $tempo_{R}:T_{R}\to Q_{{+}_{RT}}\cup \left\{0\right\}$ is a function that defines the timings associated with the transitions.
  (39) $$E=\left\{Ed^{1},Ed^{2},\;Ed^{3}\right\}\cup \left\{e\right\}$$
  is the set of external events.

The Sync application in Definition (1) is a function from the set of discrete disassembly transitions to the set of external events joined with the neutral element e,

(40) $$Sync\;:\left\{T_{2},\;T_{3},\;T_{4}\right\}\to \left\{Ed^{1},\;Ed^{2},Ed^{3}\right\}\cup \left\{e\right\},$$

(41) $$Sync\;1\_R:T_{2}\to \left\{Ed^{1}\right\},$$

(42) $$Sync\;2\_R:T_{3}\to \left\{Ed^{2}\right\},$$

(43) $$Sync\;3\_R:T_{4}\to \left\{Ed^{3}\right\}$$

- $Ed^{1}=Sync\;1\_Rsignal$ is the synchronization signal for: END WP assembly of WP1 or WP2.
- $Ed^{2}=Sync2\_Rsignal$ is the synchronization signal for: END WP disassembly of WP1 or WP2.
- $Ed^{3}=Sync3\_Rsignal$$Ed^{3}=Sync3\_Rsignal$ is the synchronization signal for: END replacement of WP cylinders of WP1 or WP2.

**Figure 12:**
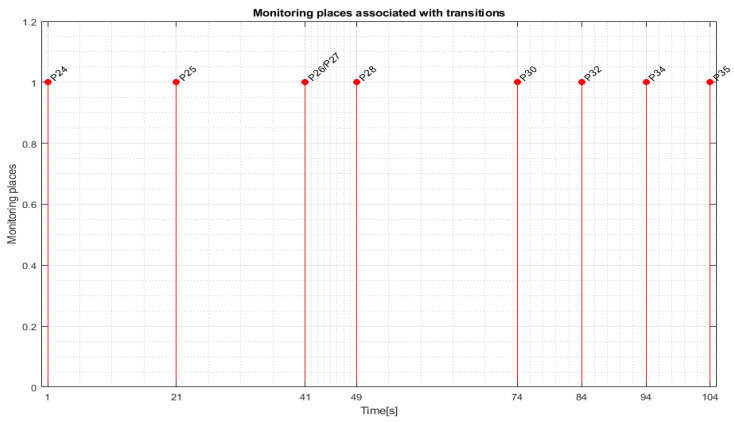
Sirphyco simulation of the STPN model for replacing one cylinder.

**Figure 13:**
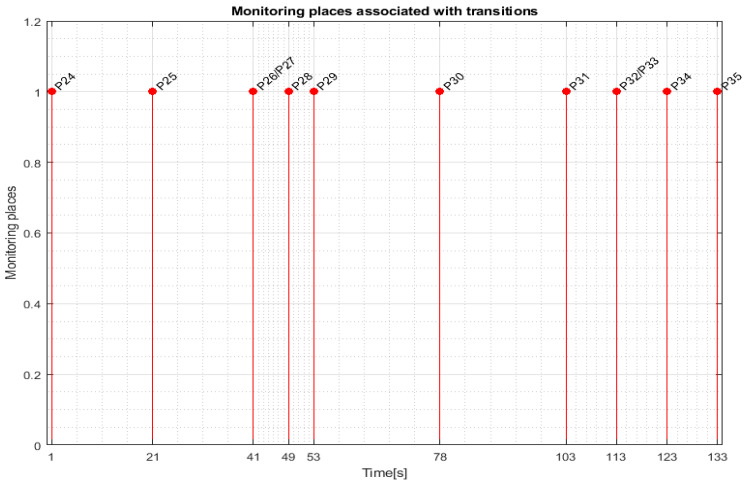
Sirphyco simulation of the STPN model for replacing both cylinders.

Transition monitoring states obtained by the STPN model simulation in Sirphyco, for the cylinder replacement of WP1 or WP2, are presented in Figure 12, for the replacement of one cylinder, and in Figure 13, for the replacement of both cylinders. As a result of the simulation of the STPN model, it can be seen in Figure 12 and Figure 13 that the monitoring states receive tokens at a time interval that corresponds to the initiation of the cylinder replacement function together with the time interval associated with the current transition.

## 4. IoT-Cloud and VPN Remote Monitoring and Control

### 4.1. Remote or Local Initialization and Selection via A/D/R HMIs

Using remote or local HMI, SCADA system, OPC-UA, interactive task planning, and the STPN models as the virtual counterpart of a digital twin, an application for the remote or local monitoring and control of three flexible A/D/R technologies on the MRC, was implemented. The MRC is designed in a multilevel and decentralized architecture, with the devices connected into LANs and Internet, into WAN. The software packages used make the structure and functionality of the entire system be considered as a cyber-physical system. At both HMIs, remote or local, there are initialization, selection, and start screens, designed in TIA portal, V17 [31]. 

Through the scanning process with the 3D and VPU cameras, it is possible to select the type of workpiece, WP1 or WP2, to be assembled or the material of the cylinders, metal or plastic, to be replaced. After each assembly operation, the number of components in each storage warehouse is updated. After each completion of functionality, assembly, disassembly, and replacement of cylinders, the warehouses are updated. The workpiece, assembled or with replaced cylinders, is delivered on the conveyor belt to the right exit of the MRC, corresponding to the front view. The workpieces for disassembly or cylinder replacement enter the conveyor belt from the right side of MRC (the right side of the front view of the MRC, Figure 2).

### 4.2. Real-Time Remote or Local SCADA Monitoring and Control of A/D/R MRC

**Figure 14:**
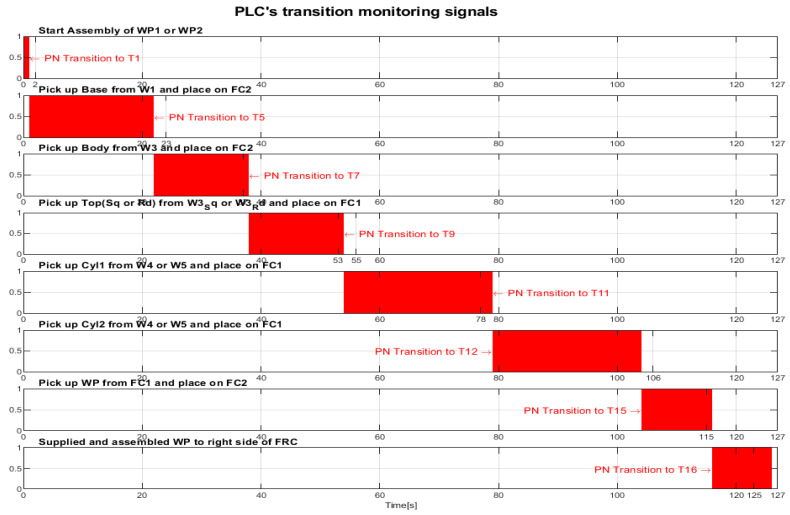
Monitoring signals (flanking transitions) from the PLC for assembly.

**Figure 15:**
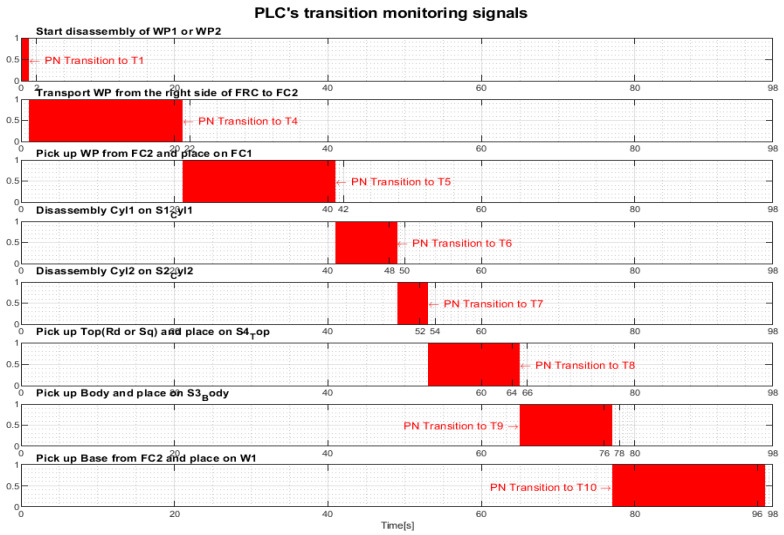
Monitoring signals (flanking transitions) from the PLC for disassembly.

**Figure 16:**
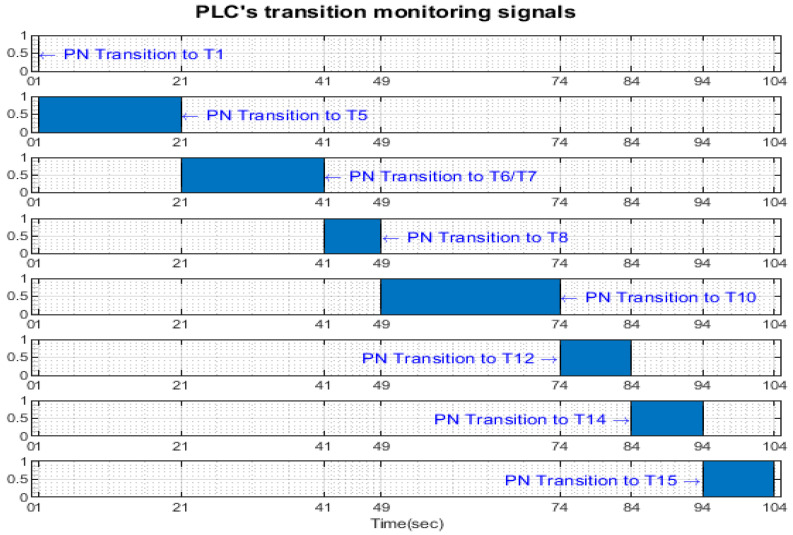
Monitoring signals (flanking transitions) from the PLC for replacing one cylinder.

**Figure 17:**
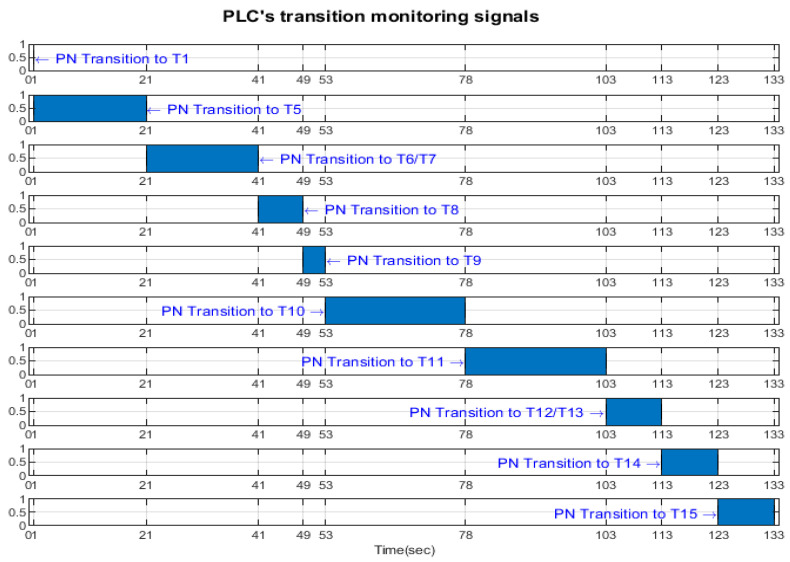
Monitoring signals (flanking transitions) from the PLC for replacing both cylinders.

After implementation, some results of the real-time control A/D/R MRC are shown in Figure 14, Figure 15, Figure 16 and Figure 17 and are compared with the monitoring signals obtained by simulation in Sirphyco, of the continuous and discrete states of the corresponding STPN models, as shown in Figure 8, Figure 10, Figure 12 and Figure 13.

The monitoring signals of the successive actions, acquired in real-time from the PLC, are represented in Figure 14 for assembly, in Figure 15 for disassembly, in Figure 16 for the replacement of a single cylinder, and in Figure 17 for the replacement of both cylinders. For comparison with the data obtained by simulation in Sirphyco, Figure 10 for assembly, Figure 11 for disassembly, Figure 12 for the replacement of a single cylinder, and Figure 13 for the replacement of both cylinders, the signals acquired in real time approximately match those in the simulation [31]. Thus, the virtual digital counterpart of the DT for A/D/R MRC is validated in real time. 

**Figure 18:**
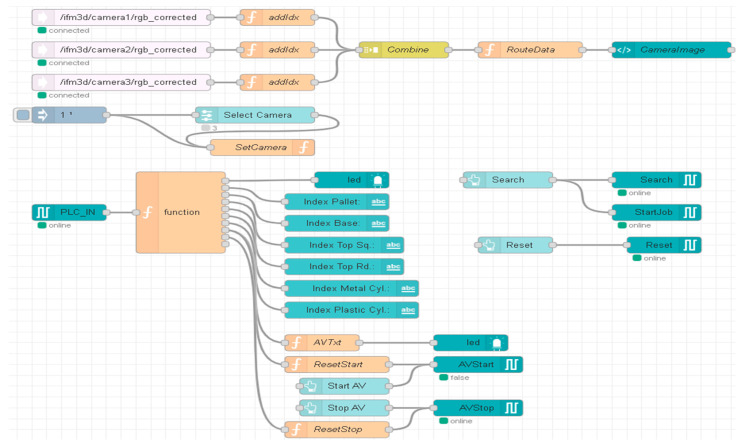
The Node-RED flow for the images captured from cameras: warehouses and parts.

**Figure 19:**
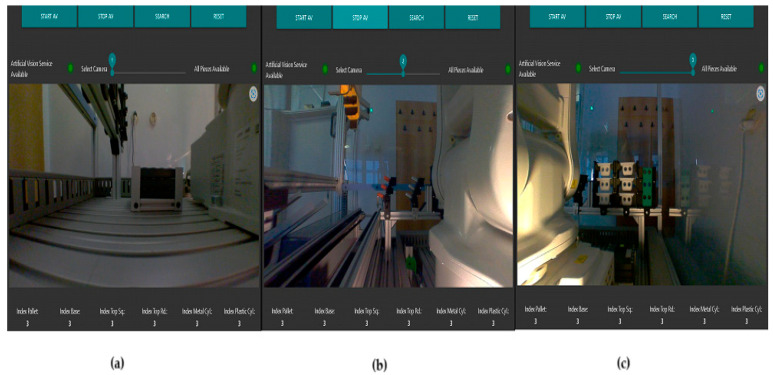
The Node-RED images captured from cameras; (**a**) warehouse with pallets; (**b**) the warehouse with metal cylinders and the one with plastic cylinders; (**c**) warehouses with bodies, with tops with square edges (Top_sq), and with tops with round edges (Top_rd), respectively.

Using the Node-RED functions, and VNC Viewer, the A/D/R operations are monitored on the remote SCADA system, in real time. Using Node-RED functions, the flow in Figure 18 was elaborated to visualize the warehouses with components, as can be seen in Figure 19. The processing of these images in the OVP800 device allows for updating the number of components in each warehouse after performing component manipulation by ABB IRM. 

### 4.3. Cloud and Embedded Computer-Based Data Storage and Analytics

**Figure 20:**
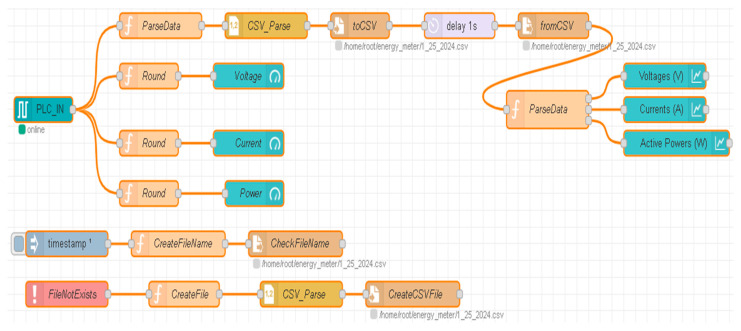
The Node-RED flow for displaying and storing electrical data of the MRC.

**Figure 21:**
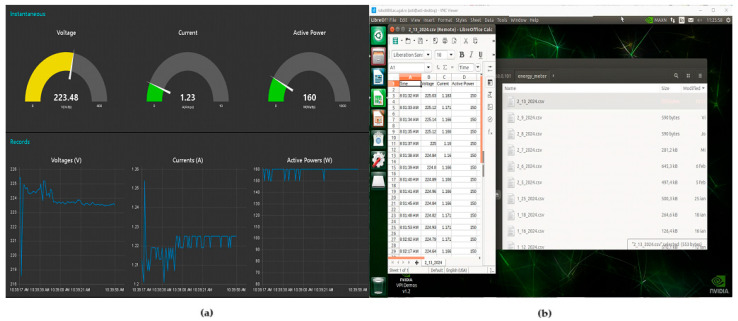
(**a**) Representation of gouge (instantaneous) and plot (records) of electrical data from the MRC; (**b**) The Virtual Network Computing (VNC)-Viewer MRC’s electrical recorded data in the embedded computer (edge device).

Through the specialized communication device, IoT 2050, Node-RED flow (Figure 20) and Virtual Network Computing (VNC), instantaneous and time-horizon electrical data, such as current, voltage, and power (Figure 21a), can be acquired and stored into the cloud and on the embedded computer (Figure 21b) for remote and local monitoring, respectively, which is useful for the prevention of breakdowns and maintenance. The ABB 120 IRM Teach Pendant (4A in Figure 1) has a VNC server installed where the user can connect remotely using the VNC Viewer desktop application. The NVIDIA Jetson Nano also has a VNC server running on it, and the user can access this server to work on it remotely if needed. The NVIDIA Jetson Nano is a small, powerful computer for embedded AI-ML applications. IoT 2050 delivers more power communication between the embedded computer and the remote PC (A/D/R Display device, 9 in Figure 1) and the cloud platform. However, since communication is wireless, the speed may not be good, causing the VNC window freeze. Due to this reason, to increase speed of communication, it is recommended to connect with an Ethernet cable to the Siemens Simatic SCALANCE XB005 switch, on the last available port, LAN P2. Therefore, we set a static IP address on the local PC [32,33,34,35].

### 4.4. Machine Learning for Adaptive Control, Optimization, and Predictive Maintenance

A machine learning algorithm processes the electrical data acquired from the robotic system, power consumption, motor current, and voltage levels (from the motors in the joints of ABB IRM and the motor that drives the conveyor belt) to create an adaptive model, with parameters updated online (gradient update law), that optimizes the system’s performance. The system learns from historical data to improve energy efficiency, reduce wear on mechanical parts, and adjust control strategies in real time. AI-based machine learning techniques are used to detect anomalies in electrical parameters, helping prevent malfunctions or unexpected downtime. By continuously learning from operational data, the system can predict faults and recommend corrective actions before they impact productivity.

### 4.5. Type-3 Fuzzy Logic and Fractional-Order Learning in the Context of AI

Type-3 fuzzy logic and fractional-order learning offer a convincing direction for enhancing the control and adaptability of A/D/R MRC [36,37]. Type - fuzzy logic enhances decision-making in dynamic environments by increasing robustness to uncertainty. Type--3 fuzzy logic, with its ability to handle higher levels of uncertainty, would be valuable for dealing with the complex, interdependent data from IoT-cloud networks, remote VPN access, and predictive maintenance in machine learning-based monitoring. With adaptive responses in real time, by integrating type-3 fuzzy logic, the A/D/R MMRC can better handle fluctuations in real-time data from the MRC and virtual simulations, potentially improving accuracy in task execution (assembly/disassembly/replacing) and enhancing resilience to variability in sensor data.

Fractional-order learning, for flexible and efficient control, optimizes control for nonlinear dynamics. It can manage the nonlinear behavior in the MRC with more precision than conventional learning algorithms. In tasks involving nuanced adjustments (such as A/D/R precision or real-time monitoring adjustments), fractional-order control provides finer tuning and reduces overshoot, which is especially important for high degrees of freedom in the ABB 120 IRM. Additionally, it enhances learning speed and convergence by accelerating convergence in machine learning models used for predictive monitoring and control, making remote and local responses faster and more efficient. Fractional-order learning has integrative potential with the current multilevel architecture, improving task planning and simulation accuracy by combining with DT, AR and VR task planning, and STPN simulation. These approaches could improve the fidelity of simulations, especially in the VR environments where real-time adjustments are critical. With type-3 fuzzy logic and fractional-order learning, SCADA and HMI interactions can achieve smoother transitions between control states, ultimately leading to a safer and more stable user experience. Adding these approaches can provide both industrial and educational benefits, supporting the vision of Industry 4.0 and Education 5.0 by offering more accurate, adaptive, and intelligent control capabilities. This novel direction would also open up research opportunities in advanced control techniques for multifunctional robotic applications.

## 5. Discussion

The IoT-cloud platform enables real-time monitoring and control of the A/D/R MRC by integrating sensors, control systems, and data streams across the cloud. Additionally, IoT-cloud platform allows remote access to real-time data and control capabilities, data analytics for optimized task scheduling, diagnostics, and cloud storage for historical data and predictive analysis. VPN ensures secure communication between the control center and the robotic system, allowing authorized users to access and control the system remotely while maintaining data privacy and providing support and troubleshooting the system from any location. The DT integrates with AR, offering users an enhanced view of the MRC in real-world settings. AR is used to visualize and simulate A/D/R tasks, provide real-time instructions and feedback on task performance, optimize task sequences, and reduce errors through visual aids. VR, combined with STPN, provides a simulated environment for modeling task workflows and system operations. This allows users or students to experience the system virtually, exploring the behavior of the robotic cell across various task scenarios. Analyze task timings and dependencies using STPN to identify workflow optimizations. The HMI dashboards serve as the primary user interface for interacting with the system, displaying real-time data on ABB IRM movements, task progress, and system status. Additionally, the HMI dashboards enable the manual control of task workflows and real-time visualization of key performance indicators. The SCADA system oversees the MRC operations, allowing data acquisition from sensors and devices, real-time monitoring and alarm control, and historical data logging for performance analysis. OPC UA is the backbone of the system’s communication architecture, ensuring seamless data exchange between the ABB IRM, IoT edge devices, and the control system. This standard protocol provides cross-platform interoperability between devices, efficient and reliable data sharing for control and monitoring tasks, and easy integration of IoT edge devices into the system.

To evaluate effectiveness, we can apply a series of performance, security, usability, and reliability metrics. Each technology, assembly/disassembly/replacing, on the MRC has unique factors to assess, especially within the context of AI, Industry and Education 4.0/5.0, where interoperability, efficiency, and security are critical, as follows:

Performance monitoring by continuous tracking of latency, response time, and accuracy metrics; usability testing and user feedback by regular surveys and feedback mechanisms for users and trainees to ensure ease of use and educational effectiveness; security audits by routine security assessments, including vulnerability scans, penetration tests, and model integrity checks for machine learning; reliability testing, stress testing, and redundancy checks to ensure system resilience and robustness; periodic comparisons of system performance, accuracy, and quality metrics before and after technology implementation to evaluate tangible benefits.

To improve the design and user experience of human–machine interfaces (both HMIs for process initialization and for AR task planning) for this A/D/R MRC system, it is essential to focus on intuitive design, usability testing, and continuous user feedback, as follows: Present information in a clear format for SCADA, monitoring, and control HMIs; prioritize critical data visibility and minimize unnecessary details that could overwhelm the user; consistency across interfaces, ensuring that all interfaces (HMIs, AR, VR, SCADA) use consistent icons, colour schemes, and terminology (HMIs have been designed in TIA V17), reducing cognitive load and allowing users to transition smoothly between system components; feedback and error handling provide clear feedback for user actions and design error messages that guide users to solve the issue; for DT and AR-based task planning, it must ensure that task instructions are easy to follow, and highlight critical steps with clear visual cues; in VR simulations with STPN models, it must reduce latency to ensure a seamless, immersive experience for users. Avoid abrupt transitions, which can disrupt the sense of continuity in task planning; design dashboards that adapt to users’ roles and preferences; AI-driven assistance by integrating AI to offer contextual assistance and predictive insights (machine learning algorithms could suggest actions or alert users to anomalies, enhancing the efficiency of remote monitoring and control, and measuring error rates and accuracy in task completion, a decrease in these metrics indicates a more intuitive HMIs); reduced downtime or interruptions resulting from user error signifies improved HMI design and user training effectiveness; and design for diverse skill levels, languages, and accessibility needs, supporting Industry 4.0/5.0 inclusivity goals.

For the A/D/R MRC, being a laboratory mechatronic system, the cyber security risks span various areas, from IoT to cloud storage, digital twins, and machine learning. To eliminate or at least mitigate specific cyber security risks, together with some action to improve security, we can implement the following: IoT-cloud integration data encryption in transit and at rest in the cloud; anomaly detection by monitoring systems to detect unusual traffic patterns or device behavior that could signal a compromise; regular security audits and penetration testing; perform periodic audits and penetration tests to identify vulnerabilities across all components; security policies and training; establish robust cybersecurity policies, including strict password policies, multifactor authentication, regular training, and awareness for staff and students; develop and test an incident response plan to quickly detect, contain, and recover from cyber incidents; network segmentation to isolate sensitive systems (like SCADA, OPC UA, and ML environments) and implement micro-segmentation to further restrict communication within segments. AI-based threat detection can be implemented with AI-driven anomaly detection systems that analyze patterns across network traffic, device behavior, and system logs to detect unusual activity early; regularly backing up critical data and configurations, and testing disaster recovery procedures to ensure rapid system restoration after an incident. By implementing these security controls and best practices, you can mitigate the risks associated with integrating IoT, cloud, VPN, DT, AR, VR, SCADA, OPC UA, and ML into your laboratory mechatronic system, such as A/D/R MRC. These steps will bolster security across the entire infrastructure and create a resilient environment aligned with Industry 4.0 and 5.0 goals in a secure and efficient manner.

## 6. Conclusions

This advanced system combines multiple technologies to enable remote and local monitoring, task planning, and control of an A/D/R MRC. The A/D/R MRC is centered around a 6-DOF ABB 120 IRM, designed for A/D/R operations in both industry and educational settings. The system incorporates modern technologies, including IoT-cloud, VPN, DT, AR, VR with STPN, HMI, SCADA, OPC UA, and an ML method (adaptive, type-3 fuzzy logic and fractional-order learning) for electrical data learning, or aligning with Industry and Education 4.0 and 5.0 principles. The integration of IoT-cloud, ML, real-time monitoring and control aligns with Industry 4.0, where the focus is on the automation and optimization of industrial systems. The system provides full remote control, enabling real-time decision-making and automated adjustments based on predictive analytics and maintenance. Instead, Industry 5.0, considered an extension of Industry 4.0, emphasizes collaboration between humans and machines. The system’s use of augmented and virtual reality, as well as MLs, allows users to work closely with robotic systems, offering personalized and human-centric interactions. This emphasizes collaboration between humans and machines.

Thus, the contributions and results obtained, up to this point, are the following:

- Multilevel architecture, hardware, and software setup.
- DT approach based on AR for task planning and VR with STPN models, formalism, and simulation. DT, coupled with AR, creates a dynamic, real-time virtual model that mirrors the physical 6-DOF ABB IRM robot and associated mechatronic systems. This model enables precise task planning, simulation, and adjustment, which is crucial for A/D/R tasks and complex, multifunctional robotic operations.
- Remote and local HMI, SCADA, OPC-UA and cloud platform. Combining cloud and VPN technologies with IoT for secure and flexible remote and local control allows seamless connectivity and robust cybersecurity. This multi-layered setup enables remote access and monitoring, which are not only efficient but also enhance cybersecurity for critical infrastructure. Integrating SCADA, HMI, OPC UA, and IoT data collection allows real-time and reliable control of the entire system, covering remote cloud access to local HMI interfaces.
- STPN simulation within VR offers a novel, structured approach to model and analyse robotic cell tasks (A/D/R) in real-time, reflecting the actual process dynamics and timing constraints of an Industry 4.0 setting. STPN provides accurate, timed sequences within VR, creating a high-fidelity environment for training and process optimization. This is especially valuable in Education 5.0, where experiential learning is critical, allowing students and operators to gain real-time, hands-on experience with industrial systems.
- Real-time monitoring and control of A/D/R MRC.
- Self-optimizing and adaptive control by ML that enables the system to adapt to operational conditions, enhancing performance over time by learning from data generated during task execution. This is particularly innovative in robotic cells where continuous improvement of task efficiency and precision is essential.
- Predictive maintenance and anomaly detection by analysing historical and real-time data, ML algorithms detect potential failures or inefficiencies before they occur, reducing downtime and improving safety and operational continuity. Electrical data acquisition, remote and local storage for preventive maintenance by adaptive ML.
- Statement of the compatibilities of A/D/R MRC with Industry and Education 4.0 and 5.0. User-centered HMI design to be accessible and intuitive for both experienced operators and students, supporting real-time system control and monitoring. This usability focus reflects the system’s dual purpose for both industrial and educational environments, aligning with Education 5.0’s emphasis on hands-on, intuitive learning. The SCADA system is fully accessible across platforms, allowing users to monitor the system remotely or locally, facilitating easy access to operational data and real-time control.
- By using OPC UA as a unified communication protocol, the system is highly interoperable, allowing easy integration of additional components or other control systems in the future. This aligns with Industry 4.0 principles, enabling scalability and flexibility. The choice of interoperable protocols and modular design enables the system to integrate new technologies and adapt to future Industry 5.0 demands with minimal reconfiguration.
- Through VR and AR simulations, students can safely practice complex tasks (assembly, disassembly, replacing, and maintenance) virtually before working on the physical robotic cell. This hands-on experience aligns with Education 5.0’s emphasis on preparing students for Industry 4.0 careers by integrating advanced technologies into learning.
- Security mechanisms and anomaly detection, especially for sensitive data (e.g., user inputs, machine learning data), improve resilience against cyber threats. Such a layered cybersecurity approach is essential for safeguarding Industry 4.0 and Education 5.0 technologies.

In the context of AI and Industry 4.0/5.0, in the future, the research focuses on the following:

- Integration of AI to improve the decision-making process.
- AI-driven predictive analytics for identifying potential issues and optimizing task planning and real-time machine learning to adapt control strategies based on the data generated by the MRC.
- AI integration with DT for simulating different operational scenarios and identifying the most efficient workflows.
- Remote learning opportunities where students can interact with the system through AR, VR, and DTs.
- Hands-on experience with real-world technologies, such as ML, SCADA, OPC UA, and IoT, in a controlled laboratory setting.
- Collaborative learning platforms where students can experiment with the system from anywhere, preparing them for the future of smart manufacturing.

Finally, the A/D/R MRC system combines cutting-edge technologies, such as digital twins with AR, VR with synchronized TPN, and machine learning-driven predictive control, to create a multifunctional robotic cell that can be used in both industrial and educational settings. Its high level of interoperability, user-centred HMI, secure remote and local access, and focus on experiential learning in Education 5.0 highlights its uniqueness. This approach bridges the gap between theoretical learning and practical industry applications, providing a robust, flexible, and scalable solution tailored to meet the evolving demands of Industry 4.0 and Education 4.0/5.0. The proposed system is a novel integration of several advanced technologies, creating a unique and forward-looking laboratory mechatronic system with real-world applications in manufacturing, robotics, and education. Above was an outline of the novelty and innovation behind this system and approach, especially in the context of Industry 4.0 and Education 5.0.

## Data Availability

Data availability is not applicable to this article, as the study did not report any data.
